# Distinct oculomotor signatures for task disengagement and reduction in vigilance during a supervisory task

**DOI:** 10.3389/fnrgo.2026.1706491

**Published:** 2026-05-15

**Authors:** Stefania C. Ficarella, Nicolas Maille, Nicolas Lantos, Kevin Le Goff, Jean-François Sciabica, Jean-Christophe Sarrazin, Andrea Desantis

**Affiliations:** 1 Information Processing and Systems Department, Cognitive Engineering and Applied Neuroscience, ONERA, Salon-de-Provence, France; 2 Institut de Neurosciences de la Timone, CNRS & Aix-Marseille Université, Marseille, France; 3 Fédération ENAC ISAE-SUPAERO ONERA, Université de Toulouse, Toulouse, France; 4 Airbus Operations SAS, Toulouse, France

**Keywords:** eye-tracking, out-of-the-loop (OOTL), task engagement, task monitoring, vigilance

## Abstract

Technological advancements in human-machine interfaces increasingly confine human operators to the role of passive supervisors. Under such conditions, the out-of-the-loop phenomenon, driven by vigilance decline and task disengagement, can result in degraded performance, when automated systems fail unexpectedly. Despite decades of research on the topic, characterizing, quantifying and predicting these performance problems remain difficult. This study aimed at identifying distinct oculomotor signatures associated with task disengagement and vigilance reduction during a specifically designed supervisory task. Participants viewed a simplified aircraft interface and were asked to monitor an autopilot system and validate or reject its decisions. Task disengagement was manipulated by varying the time delay before participants were required to intervene: in some cases, responses were frequent, while in others participants had to wait longer before having to intervene. Vigilance was manipulated through time-on- task. Results showed that participants' performance was negatively impacted by task disengagement (slower reaction times when less solicited). Although visual exploration decreased both under low vigilance and low engagement, other oculomotor markers (i.e., saccades, fixations, eyelid opening and blinks) could dissociate the two states. Finally, logistic classifiers revealed that specific oculomotor features predicted participants' reaction times above chance. In summary, this study identified distinct oculomotor patterns linked to vigilance decline and task disengagement, two factors contributing to the out-of-the-loop phenomenon, which were also predictive of individuals' performance. These results are not only relevant for the monitoring of piloting activities, but contribute to the general knowledge of the impact of task disengagement on human performance.

## Introduction

Technological advancements in transportation and industrial domains lead to increasingly more automated machines that ensure task proficiency, while simplifying the role of human operators (Baxter et al., 2012; Cummings et al., 2010; Naujoks et al., 2016; Parasuraman, 1987; Parasuraman and Riley, 1997; Wise et al., 1994). However, the substitution of human activity with tasks performed by automated machines is far from being a trivial operation (cf. substitution myth Dekker and Woods, 2002; Woods and Tinapple, 1999). In fact, automation alters the cooperative architecture, changing the role of humans in profound ways and creating new problems for operators (Sarter et al., 1997). More specifically, automation shifts the role of operators from active controllers to passive supervisors of efficient automated systems that very rarely fail, thus demanding very few, but critically important, human interventions (“automation-induced complacency,” see Parasuraman et al., 1993). This shift in the role of operators impacts their ability to predict automated systems' decisions and action consequences and, ultimately, their ability to process new information (Parasuraman and Wickens, 2008).

This series of impairments are known as the out-of-the-loop performance problem, and they can lead to serious safety issues (Berberian et al., 2017; Billings, 1991; Endsley, 1996; Endsley and Kiris, 1995; Kaber and Endsley, 2004). For instance, in case of failure of the automated system, operators may have troubles understanding the source of the problem and selecting the appropriate actions to solve it. These automation surprises (Sarter et al., 1997) are particularly well-documented (Degani and Heyamann, 2000) and can lead to serious performance impairments, which may have critical consequences, especially in the aviation domain (Bureau d'Enquête et d'Analyse, 2012a, p. 10, Bureau d'Enquête et d'Analyse, 2016, p. 44; Bureau d'Enquête et d'Analyse, 2012b, p. 178; Bureau d'Enquête et d'Analyse, 2002, p. 167; National Transport Safety Board, 1972, p. 17). Hence, investigating the sensory, cognitive, and motor functions of operators affected by the interaction with highly automated systems is critical to improve safety.

Despite decades of research, characterizing, quantifying and predicting the occurrence of out-of-the-loop performance problems remain difficult, particularly pinpointing the specific mental processes that are impacted (Baxter et al., 2012). The previously mentioned complacency, particularly evident for long (time-on-task) supervisions of overly reliable automated systems may lead to task disengagement. The latter refers to the reduced allocation of processing resources to the task at hand (Dehais et al., 2020; Stephens et al., 2018). Moreover, vigilance, which can be construed as the ability to stay alert and sustain attention over time, also decreases over long periods of automated systems' supervisory tasks (Amalberti, 1999; Maille et al., 2022). As such, task disengagement and vigilance are considered two, possibly interacting, factors contributing to the out-of-the-loop phenomenon (Gouraud et al., 2018).

However, it remains unclear if it is possible, through objective, quantifiable metrics, to disentangle the contributions of these factors in the generation of the out-of-the-loop effects (for a review see Pütz et al., 2024).

Time-on-task (ToT), that is the time spent performing a specific task, is classically considered an experimental manipulation useful to induce vigilance decrement (Warm et al., 2008). As ToT increases, a change in oculomotor behavior is typically found. For instance, during extended periods of eventless car simulator driving, vigilance decrement was associated with a reduction in the oculomotor behavior typically found during processing of information (i.e., reduction in blinking and ocular activity, Campagne et al., 2005). The percentage of eyelid closure is also typically associated to performance decrement found in vigilance studies (Maille et al., 2022; Dinges et al., 1998). Moreover, McIntire et al. (2014) found an increase in blink frequency and duration at the augmenting of ToT, which reduced vigilance and performance.

As for task disengagement, few recent studies have investigated the usefulness of oculomotor data, since subjective and behavioral measures are classically used for this purpose. Hamachi et al. (2022) quantified the rate of gaze samples (the average number of samples collected in a second) and gaze velocity during three cognitive tasks categorized as “passive,” “semi-active” and “active.” The authors found that the rate of gaze samples and the logarithm of gaze velocity could be useful in estimating the level of participants' task engagement during passive semi-active and active tasks, respectively. However, the authors manipulated the amount of mental workload the three tasks are supposed to elicit, which might limit the generalisability of their results to other task engagement studies (like the present one) that adopt other methods to induce task disengagement. Similarly, Fragueiro et al. (2023) used pupil dilatation measure to quantify mental workload-induced task disengagement during neurofeedback sessions.

Contrary to these previous studies, the present work aims at identifying a more complex oculomotor behavior that might disentangle (and predict) operators' task disengagement from vigilance decrement. To this aim, an *ad hoc* monitoring task was developed, in which participants were asked to monitor an autopilot system and validate or reject its decisions. Crucially, to manipulate task engagement, the frequency of participants' required interventions varied. In some situations, participants were frequently asked to validate/reject the autopilot's decisions, while in others, a long delay could occur before their intervention was needed. The rationale behind this manipulation was that the longer an operator has to wait before taking a relevant action, the more likely disengagement is to occur (e.g., task withdrawal, mind-wandering; cf. Dehais et al., 2020). Conversely, ToT was manipulated through block repetition of the same task, to manipulate vigilance. During the task, participants' response times, decision accuracy as well as oculomotor behavior were recorded.

In terms of expected results, firstly, we expected shorter reaction times and higher accuracy when participants were frequently solicited (i.e., high engagement) compared to when they had to wait longer before intervening (i.e., low engagement). Secondly, we expected to observe a specific pattern of oculomotor behavior associated to task disengagement. Visual exploration would be lower with low compared to high engagement conditions: disengagement would lead to a decrease in the number of saccades and an increase in fixation duration. This pattern could potentially be dissociated from a pattern associated to vigilance. It is well known that reduced vigilance also leads to reduced visual exploration, by reducing eyelid opening and increasing the number and duration of blinks (Kuwahara et al., 2022; Nguyen et al., 2015). However, while in most studies task disengagement and reduced vigilance may be confounded, the present paradigm was specifically designed to separate the two. We manipulated within-trial Delay to generate more or less task disengagement and explored the effect of ToT on vigilance through block repetitions. Finally, we explored whether oculomotor patterns could predict individuals' performance. This latter analysis is important as it could provide insight on oculomotor features that are predictive of disengagement and low performance.

## Materials and methods

### Participants

Twelve healthy volunteers (four male, average age = 28.10 years, SD = 2.42, min = 24, max = 31) participated in this experiment. All participants had normal or corrected-to-normal vision, and were naïve to the hypotheses under investigation. They all gave written and informed consent before participating in the experiment. This study was conducted in agreement with the requirements of the Helsinki declaration and approved by the ethics committee of Université Paris Cité.

### Apparatus

**Figure 1:**
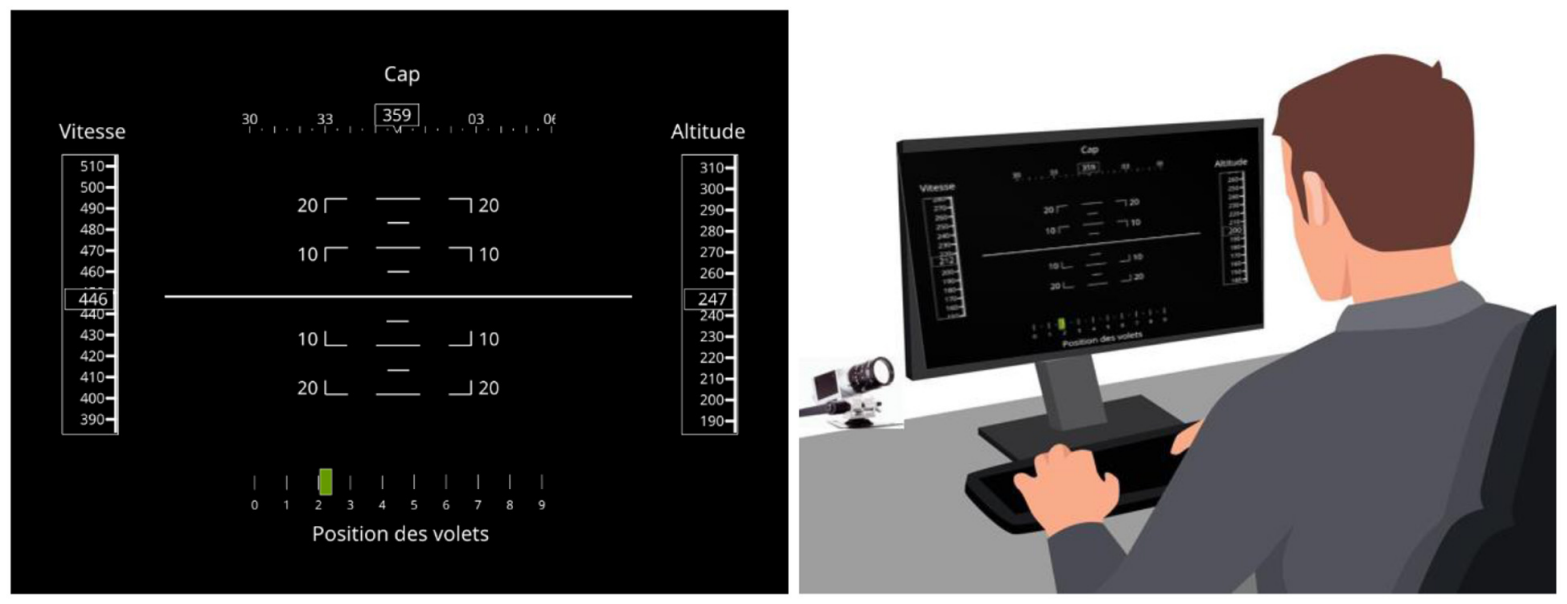
Participants were presented with a simplified aircraft interface (left panel). Throughout the task, four parameters varied dynamically [Heading (“Cap”), Speed (“Vitesse”), and Altitude]. Right panel: Participant's position during the experiment with SmartEye cameras recording the ocular movements during the task.

Stimuli were presented on a CRT LG Flatron 915FT Plus (60 Hz refresh rate) with a resolution of 1,024 × 768 pixels and a size of 36.5 × 27 cm. Stimuli presentation and data collection were performed using VAPS XT 4.2.1 (Presagis). Oculomotor data was recorded using the SmartEye Pro 3.0 (Smart Eye AB, Gothenburg, Sweden) hardware and the SmartEye 7.1.0 software (Smart Eye AB, Gothenburg, Sweden). The system included two infrared illuminators and three cameras (120 Hz sampling frequency) placed below the computer screen (see Figure 1, right panel). Gaze calibration was performed using a 4-point grid, using the Gaze Calibration Client proposed by SmartEye.

### Procedure

Participants were presented with a simplified aircraft interface featuring four parameters: aircraft altitude, speed, flap position, and heading (see Figure 1, left panel). Their task was to supervise the autopilot's activity and detect significant changes applied to the flight parameters by the autopilot. A significant change was defined as a deviation of more than 20 units for altitude, speed, and heading, and 1 unit for flaps, relative to their current values. Each deviation lasted 3 s. Once a significant change in a parameter was detected, participants compared it to the most recent significant change they had observed. If the change occurred in the same direction as the previous change (regardless of the parameter), the autopilot action was considered correct. Conversely, if the change occurred in the opposite direction (regardless of the parameter), the autopilot action was deemed incorrect. Participants responded by pressing designated keys (leftward pointing arrow if the autopilot respects the task rules, rightward pointing arrow otherwise).

**Figure 2:**
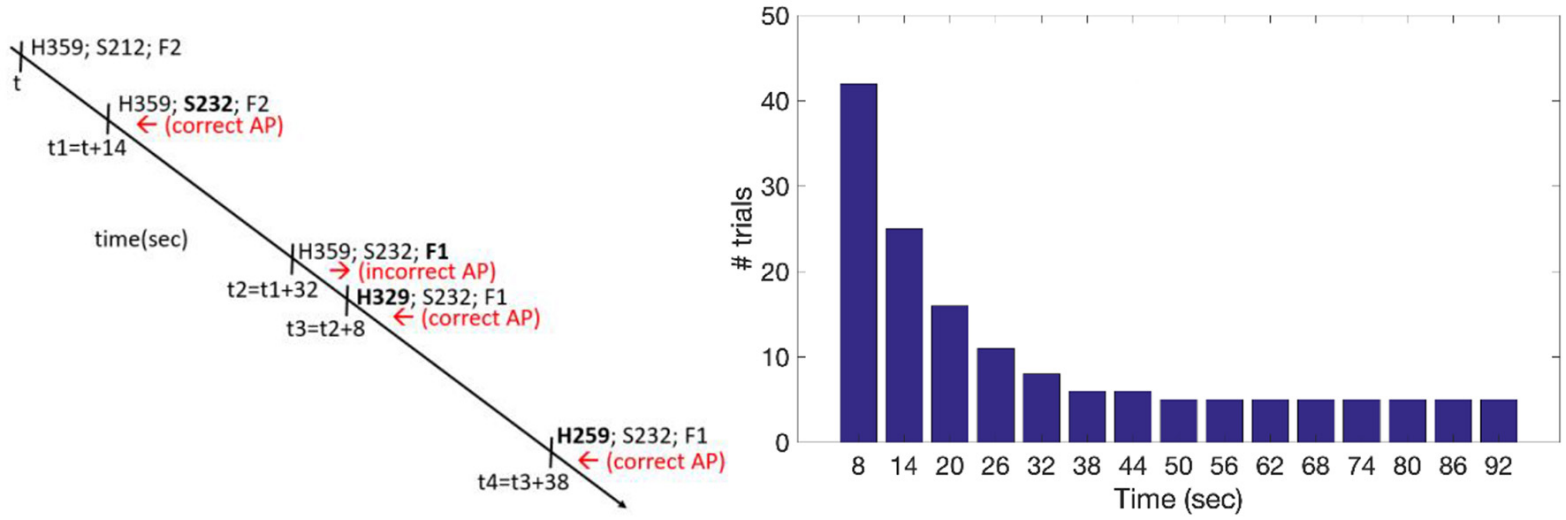
Left panel: example of significant parameters' change over time with the required responses (in red, left-pointing arrow for a correct autopilot –AP—decision and right-pointing arrow for an incorrect AP decision). Right panel: the graph depicts the relative frequency of temporal delays across the task, which were drawn from an exponential function.

For example, if altitude increased by 20 units at time “t,” and subsequently speed increased by 20 units at time “t1,” participants pressed the “correct action” key. However, if speed decreased by 20 units at time “t1,” participants had to report the autopilot's action as incorrect. The change in speed at time “t1” then served as the reference point to evaluate the change in the subsequent parameter at time “t2,” and so on (see examples in Figure 2, left panel).

To increase the difficulty of detecting significant changes in the parameters, the four parameters were not static. Background noise was introduced by varying the four parameters within a range of values from −6 to 6 for speed, altitude, and heading, and between −0.3 and 0.3 for the flap position. This variation was achieved using a combination of two sinusoidal waves: one fast at 0.15 Hz and the other slow at 0.025 Hz. Only the central display remained fixed throughout the trial, in order to encourage participants to perform eye movements.

To manipulate participants' engagement, we varied the time interval between significant changes in the parameters. Fifteen different time delays were used, ranging from 8 to 92 s, with 6 s-long increments. The frequency of presentation of each time interval throughout the task was determined using an exponential function, with intervals of 8 s being the most frequently presented. The frequency decreased as the time delay increased (see Figure 2, right panel). This manipulation was based on the underlying assumption that longer time delays would lead to greater disengagement from the monitoring task. We employed an exponential function to control for the hazard rate, which describes the conditional probability of an event occurring at a specific point in time, given that it has not yet occurred. In fact, it has been demonstrated that longer foreperiods decrease reaction times due to increased certainty of target presentation (Näätänen, 1970).

A total of 154 parameters' changes were included within the task^1^. A short break was proposed in the middle of the task. Accordingly, a total of 152 responses were collected (participants provided no response to the first parameter change at the beginning of the experiment and after the break). The average total duration of the task was 90 min.

### Data analysis

#### Behavioral data

Statistical analyses were conducted using hierarchical Bayesian models implemented with brms package (Bürkner, 2017) in R 4.4.1 (R Core Team, 2024). We used Bayesian analyses because they are more robust to convergence issues and provide stable parameter estimates even with limited data compared to classical frequentist approaches (Kruschke, 2021). In addition, the Bayesian implementation in brms offers a wide range of probability distributions that are particularly suitable for the types of variables analyzed in the present study. Accuracy (i.e., whether participants correctly reported the actions of the autopilot) was analyzed using a Bayesian logistic mixed-effects model. Response times (RTs) were measured from the onset of each significant parameter change. As RTs exhibited the typical right-skewed distribution, they were analyzed using Bayesian ex-Gaussian mixed-effects models. Trials in which no judgment was provided by the participant were removed from the analyses. In total 1,533 responses were analyzed, about 10 % of the trials did not include a response.

The primary aim of this study was to examine the effect of Delay on accuracy and RTs, as longer delays may induce operator disengagement from the monitoring task. In these analyses, Delay was treated as a continuous predictor. The models also included Block (10 blocks) as an additional continuous predictor. This variable reflected different moment of time within the task: the first block corresponded to the first 15 responses and the last block to the last 15 responses for each participant. This predictor was included to account for potential vigilance effects over time.

To account for the well-known relationship between accuracy and RTs, an additional predictor was included in each model. Specifically, in the accuracy analyses, RT (treated as a continuous variable) was entered as a covariate, whereas in the RT analyses, accuracy (correct vs. incorrect) was included as a factor. Models including by-participant random slopes and intercept were tested. However, given the relatively small number of participants, estimating random slope variance components is challenging and may lead to unstable or poorly identified models. In such situations, the data provide limited information to reliably estimate between-participant variability in slopes. Hence, these models were compared to simpler and more parsimonious models including only by participants random intercepts, using a leave-one-out cross-validation. The comparison showed the model including random slopes for delay and block did not meaningfully improve predictive performance relative to the simpler model with only by-participant random intercepts, while substantially increasing model complexity. Hence, the final models retained for the analyses included only by-participant random intercepts. Further details regarding models and these comparisons can be found in the Supplementary material.

Accordingly, the model structures were as follows: rt ~ delay + block + accuracy + (1|subject) and accuracy ~ delay + block + rt + (1|subject). Delay, Block, and RTs were mean-centered across participants. To evaluate the presence of effects, parameters were classified as showing strong evidence for an effect when 95% Credible Intervals (CrI) excluded zero. This heuristic follows the interpretation of the probability of direction proposed by (Makowski et al. 2019). For all Bayesian models, convergence was evaluated using R-hat statistics (< 1.01) and effective sample sizes (ESS), and overall model adequacy was assessed using posterior predictive checks to ensure that the fitted models captured the key features of the empirical distributions. Full model summaries are reported in the Supplementary material, including parameter estimates (Estimate, Estimate Error, lower and upper 95% credible intervals), convergence diagnostics (R-hat, Bulk ESS, Tail ESS), and posterior predictive check plots for each model.

#### Eye-tracking data

Regarding the eye-tracking data, we were particularly interested in internally driven oculomotor behavior and how it relates to individuals' performance and engagement (as manipulated through the delay between interventions). For this purpose, we extracted 4-s segments ranging from −4 s to 0 s, time-locked to the onset of each parameter change. We selected this time window because it ensured that the sampled segments always occurred during periods in which no parameter change was present and only background noise was displayed. This allowed us to isolate eye movements that were internally driven rather than stimulus-driven.

The shortest delay between two parameter changes in the experiment was 8 s, and each parameter change lasted 3 s, leaving a consistent 5-s interval in every trial during which the display was driven solely by background noise. We excluded the first second of this interval to avoid any residual attentional capture from the preceding parameter change. This yielded a consistent 4s, stimulus-free window that was well-suited for analyzing internally driven oculomotor behavior.

Within these segments, we extracted several oculomotor features, including the number of fixations, total fixation duration (i.e., the sum of all fixation durations), number of saccades, number of blinks and their total duration, and eyelid opening. These features were calculated directly by the Smart Eye software using the built-in event-classification algorithms implemented in the Smart Eye. The Smart Eye software provides also quality check measures for pupil dilation and eyelid opening. Since pupil dilation was not analyzed in this experiment, we quantified the eyelid opening signal quality using the Smart Eye measurement within the −4 to 0 s window. Signal quality was high across participants (mean = 0.89, SD = 0.07, range = 0.74–0.99). The proportion of valid samples was 100% for all participants, and no trials contained windows without valid eyelid-opening samples.

To analyse these features, we employed Hierarchical Bayesian mixed models (see behavioral data analyses). As for behavioral analyses, all eye-tracking models included Delay and Block as continuous predictors and by-participant random intercepts, i.e., eye variable ~ delay + block + (1|subject). Block was included to control for potential changes in vigilance or fatigue across the experiment, which are known to influence oculomotor behavior. We decided to report only models including by-participants random intercepts, since, given the relatively small sample size, estimating random slope variance components is challenging and may lead to unstable or poorly identified models. However, in the Supplementary material we report also results with random intercepts and random slops, at least only in the cases where including random slopes did not produce substantial sampling pathologies (divergent transitions and poor energy diagnostics). Overall, the results with or without random slopes were very similar.

The distributions of the number of fixations, blinks, and saccades were positively skewed, which is typical for count data reflecting the number of discrete oculomotor events per trial. Such variables are appropriately modeled using discrete probability distributions such as Poisson or negative binomial, rather than Gaussian models. Fixation counts exhibited approximate equidispersion (variance close to the mean), and the Poisson distribution provided stable estimation and excellent posterior predictive fit. We therefore modeled fixation counts using a Poisson regression. Blink counts showed many zero blink trials and a rapidly decreasing number of trials with 1–3 blinks. Although overall dispersion was modest, the excess zeros warranted testing for zero inflation. A zero-inflated Poisson model provided the best fit, capturing both the high proportion of structural zeros and the distribution of non-zero blink counts. In contrast, saccade counts showed clear overdispersion (variance substantially larger than the mean) and a highly skewed distribution. Poisson models failed to account for this variability, whereas the negative binomial distribution, which includes a dispersion parameter, provided a markedly better fit and realistic simulated data.

Fixation duration, blink duration, and eyelid opening were continuous, strictly positive variables that showed mild deviations from normality, with a small number of extreme values. To stabilize variance and improve symmetry, all three measures were log-transformed prior to analysis. The transformed variables were then modeled using a Student-*t* likelihood, which provides robustness to outliers and accommodates both left- and right-tailed deviations from normality better than Gaussian or lognormal alternatives.

#### Classification analyses

Further analyses explored the extent to which oculomotor features can predict individuals' performance, specifically response times. Logistic regression classifiers were trained and tested using different oculomotor features to differentiate between fast and slow response times for each participant across three different time periods. Slow and fast response times were defined as responses slower and faster than the median response time (±1/3 of the median absolute deviation), respectively. The oculomotor features used to predict response times included: the number and duration of saccades, the number and duration of fixations, the number and duration of blinks, pupil size, and eyelid opening. Instead of working with 4-s time windows as with the mixed model above, we decided to increase the time resolution of the classification analyses, by dividing the 4-s segments into two separate time-bins and by adding 2 s prior to that time window (i.e., from −6 to −4 s prior to the parameter change) even though that time period might contain stimulus-driven ocular activity. Hence, oculomotor features were calculated for three different time periods: from −6 to−4 s, from −4 to −2 s, and from −2 to 0 s prior to the onset of the parameters. A separate classifier was trained and tested in each specific time window. Note that we also tested a classifier using a single 4-s time window, matching the time window used in the mixed-effects model analyses, which yielded similar results. The classification procedure implemented a Monte Carlo cross-validation method (Dubitzky et al., 2007). Notably, each classifier was trained on 80% of the available dataset and tested on each of the remaining observations. This procedure was repeated 1,000 times. Each time, a random 80% of trials was used as training set and the rest as test set. To avoid classification biases, at each repetition of the procedure the number of slow and fast responses were matched. Classification accuracy was estimated by calculating, for each time period, the proportion of responses that the classifier correctly identified as fast or slow. The mean classification performance of the 1,000 shuffling within each participant and time period was taken as the classifier accuracy for that specific participant and time period. For statistical analyses chance level (a probability of 0.5) was subtracted from classification accuracy. Statistical significance (α = 0.05) was then calculated using a non-parametric cluster-based permutation test. In addition to classifier accuracy, we computed the Area Under the Curve (AUC) of the Receiver Operating Characteristic (ROC) to provide a threshold-independent measure of classification performance. Mean AUC values were calculated in the same manner as classification accuracy and statistically compared against chance level (0.5) across participants with a cluster-based permutation test. AUC results are reported together with classification accuracy in the Results section, and the corresponding ROC curves are provided in the Supplementary material.

## Results

### Behavioral data

**Figure 3:**
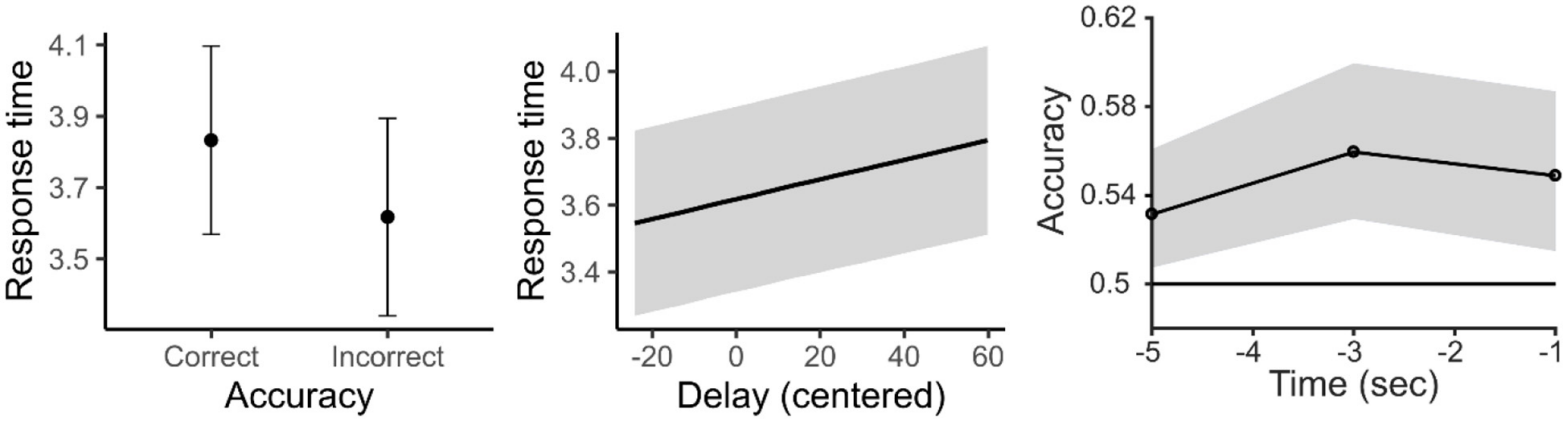
The left panel shows the posterior mean response time as a function of accuracy, with error bars indicating ±1 posterior standard error around the mean. The central panel displays the marginal posterior relationship between response time (*y*-axis) and delay (*x*-axis, centered within participants). Shaded regions represent ±1 posterior standard error around the mean. The right panel shows the accuracy (proportion of correct classification) of a logistic classifier dissociating fast and slow responses from oculomotor features. The classifier was trained and tested on oculomotor features occurring prior to the onset of significant parameter change. The horizontal black line corresponds to chance level (50%) accuracy, indicating that the three time periods selected for the analysis exhibited a classification accuracy significantly above chance level. Shaded area represents bootstrapped 95% confidence intervals.

The posterior estimates of the accuracy analyses indicated no credible effect of Delay on accuracy [β = −0.0036, 95% CrI (−0.0099, 0.0026), *P* (β < 0) = 0.8702] or of Block [β = 0.015, 95% CrI (−0.044, 0.074), *P* (β > 0) = 0.692, see Figure 3, right panel]. The effect of RT on accuracy was also small, and its credible interval included zero [β = −0.044, 95% CrI (−0.087, 0.001), *P* (β < 0) = 0.97], suggesting limited evidence for a systematic relationship between response time and accuracy. The intercept [β = 2.28, 95% CrI (1.89, 2.69)] reflects the high overall accuracy in the task (Mean = 0.90, SD = 0.30).

Posterior estimates from the reaction time analysis indicated strong evidence that RTs increased with longer delays [β = 0.0029, 95% CrI (0.00086, 0.00494), *P* (β > 0) = 0.9968], indicating that responses slowed as the interval preceding the target increased (Figure 3, central panel). A slight decrease in RT across blocks was suggested by the posterior estimates [β = −0.015, 95% CrI (−0.034, 0.002), *P* (β < 0) = 0.96], although the credible interval included zero, indicating that the evidence for this effect is weak. If present at all, this trend may reflect only a minimal familiarization as the task progressed. Accuracy also affected RTs: correct responses were slower than incorrect responses [β = 0.221, 95% CrI (0.032, 0.414), *P* (β > 0) = 0.9891], consistent with a small speed–accuracy trade-off, where responses that were more careful were likely to be more correct (Figure 3, left panel).

### Eye-tracking data

**Figure 4:**
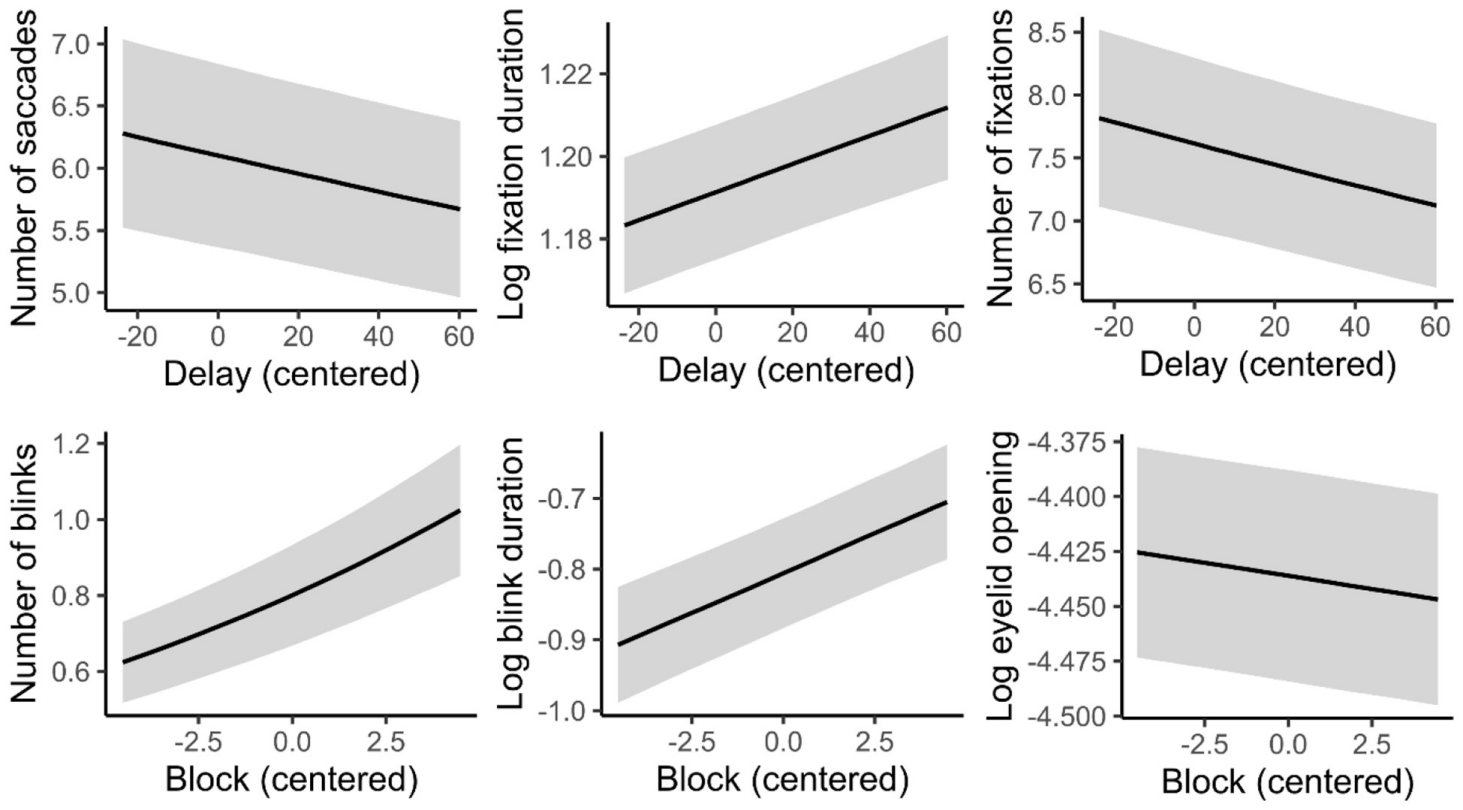
Top panel: Posterior predictions showing the relationships between the number of saccades **(left)**, fixation duration **(center)**, and the number of fixations **(right)** as a function of the parameter-change delay (*x*-axis). Bottom panel: Posterior predictions for the number of blinks **(left)**, blink duration **(center)**, and eyelid opening **(right)** as a function of time on task (block number). Shaded regions represent ±1 posterior standard error around the population-level mean prediction. Block and Delay variables are centered, with larger number indicating longer time-on-task and longer delays, respectively.

To assess whether disengagement from the supervisory task and the time-on-task affected participants' visual exploration strategies, we firstly looked at the impact of Delay and Block on the number of saccades and fixations. Posterior estimates from the negative binomial model revealed that the number of saccades decreased slightly with longer delays [β = −0.0012, 95% CrI (−0.0020, −0.00035), *P* (β < 0) = 0.9968], indicating a small but reliable reduction in saccade frequency as the interval preceding the target increased (Figure 4, top left panel). In contrast, there was no credible evidence for an effect of block [β = −0.0030, 95% CrI (−0.0106, 0.0045), *P* (β < 0) = 0.7820], suggesting that saccade counts remained stable across the course of the experiment.

Posterior estimates from the Poisson model showed that the number of fixations decreased slightly with longer delays [β = −0.0011, 95% CrI (−0.00175, −0.00047), *P* (β < 0) = 0.9997], indicating a small but reliable reduction in fixation frequency as the delay preceding the target increased (Figure 4, top right panel). There was no credible evidence for an effect of block [β = 0.0027, 95% CrI (−0.00317, 0.00856), *P* (β > 0) = 0.8211], suggesting that fixation counts remained stable across the experiment.

In the meantime, fixation durations indicated that fixations became slightly longer at longer delays [β = 0.00034, 95% CrI (0.00015, 0.00053), *P* (β > 0) = 0.9999], reflecting a small but reliable increase in fixation duration as the interval preceding the target increased (Figure 4, top central panel). In contrast, fixation durations decreased across blocks [β = −0.0070, 95% CrI (−0.00870, −0.00537), *P* (β < 0) = 1], providing strong evidence for progressively shorter fixations over the course of the experiment. Taken together, these findings indicate that the visual exploration of the interface decreased with longer delays: less saccades and fewer but longer fixations for longer time delays. While a different pattern was observed with the time spent on the task (Block number), where the duration of fixations decreased with time, while the number of fixations and saccades remained stable across time.

If fixation durations decreased over time on task, one might expect this to be accompanied by an increase in the number of saccades. However, this pattern was not observed. This suggests that other oculomotor factors may account for the change in fixation duration, such as variations in blink frequency or duration, or changes in eyelid opening, features that may themselves reflect a decline in vigilance over the course of the experiment. The analyses of the number of blinks showed that the number of blinks decreased slightly with longer delays [β = −0.00225, 95% CrI (−0.00420, −0.00035), *P* (β < 0) = 0.9893]. In contrast, blink frequency increased across blocks [β = 0.0550, 95% CrI (0.0375, 0.0722), *P* (β > 0) = 1], providing strong evidence that participants blinked more frequently as the task progressed, potentially reflecting a gradual decline in vigilance or alertness over time (Figure 4, bottom left panel). In addition, blink durations increased across blocks [β = 0.0224, 95% CrI (0.0115, 0.0336), *P* (β > 0) = 0.9999], providing consistent evidence pointing to a gradual decline in vigilance or arousal over time (Figure 4, bottom central panel). In contrast, we observed no credible effect of delay on blink duration [β = −0.00069, 95% CrI (−0.00190, 0.00052), *P* (β < 0) = 0.8672]. Finally, eyelid opening decreased across blocks [β = −0.00242, 95% CrI (−0.00384, −0.00098), *P* (β < 0) = 0.9995], corroborating the idea that participants tended to narrow their eyelids as the task progressed due to reduced arousal/vigilance or increasing fatigue (Figure 4, bottom right panel). No evidence of a change in eyelid opening as a function of delay was observed [β = −0.000056, 95% CrI (−0.00021, 0.00010), *P* (β < 0) = 0.7561]. However, note that the effect of block on eyelid opening became uncertain when modeling random slopes (see Supplementary material), suggesting that important between subject variability was observed for this feature.

In sum, these results seem to show distinct oculomotor signatures associated to disengagement and reduction of arousal. They would both lead to a reduced exploration of the visual environment. However, disengagement is characterized by fewer saccades and fewer but longer fixations, while decreased vigilance is associated with a higher number and longer duration of blinks, along with smaller eyelid opening.

Finally, additional analyses examined the extent to which oculomotor features could predict individuals' response times. Classification analyses revealed that during the entire pre-selected time period (i.e., from −6 s to 0 s prior to the onset of the parameter change), we could predict at above-chance level whether participants were going to react slowly or quickly, based on oculomotor behavior *preceding* the response (see Figure 3, right panel). The highest classification accuracy observed was 56%. Neighborhood component analysis showed that the three oculomotor features that contributed the most to classification accuracy were: the number of saccades, the duration of fixations and the duration of blinks. AUC values for each time window were as follow: Average AUC for −6 to −4 s window = 0.5411; Average AUC for −4 to −2 s window = 0.5776; Average AUC for −2 to 0 s window = 0.5679. The entire time cluster was significant *P* < 0.001. In sum, ROC analysis confirmed above-chance classification performance indicating modest but reliable discriminability.

Note that similar classification results were obtained when focusing specifically on the 4-s time window used in the mixed model analyses (*P* = 0.0103, Average Classification Accuracy = 0.549, Average AUC = 0.571, *P* = 0.0054).

## Discussion

The present research aimed at contributing to the characterization of the impact of task disengagement on human performance, at identifying an oculomotor behavior that might predict operators' disengagement, and dissociating it from oculomotor behavior associated to a reduction in vigilance. Engagement was defined here as the ability mobilize and allocate cognitive resources to a task in order to correctly react to an operational solicitation.

Engagement was manipulated here by varying the time interval between significant changes in the flight parameters, ultimately requiring participants' responses. This manipulation was based on the underlying assumption that longer time delays would lead to greater disengagement from the monitoring task. Instead, vigilance was operationalized as the time spent performing the task (i.e., block progression).

The study revealed that participants exhibited shorter reaction times when they were frequently solicited (i.e., high engagement) compared to when they had to wait longer delays before intervening (i.e., low engagement). Additionally, a specific pattern of oculomotor behavior was observed in association with task disengagement. Visual exploration decreased to a greater extent under low engagement conditions compared to high engagement conditions, characterized by a decrease in the number of saccades and an increase in fixation duration with longer delays. Importantly, this pattern of oculomotor behavior associated with disengagement was distinct from that observed with vigilance. Specifically, as the time on task increased, visual exploration was also found to decrease, but was rather characterized by a reduction in eyelid opening and an increase in the number and duration of blinks, suggesting that the time spent on task decreased general arousal (Kuwahara et al., 2022; Nguyen et al., 2015). The latter effect is in line with the results of Maille et al. (2022), who found an increase in eyelid closure as ToT increased. However, note that the effect of block in the reduction of eye opening was uncertain as it disappeared when mixed models included random slopes. This suggests that large variability was observed between participants. Further studies should consolidate this finding.

Interestingly, fixation duration showed opposite trends for disengagement and time-on-task effects. Fixations became slightly longer with increasing delay, whereas they shortened across blocks, corroborating the hypothesis that we are probably observing different underlying mechanisms. Longer delays between task-relevant events may encourage transient disengagement from active monitoring. Participants may reduce visual scanning and maintain gaze longer at a given location, which could be consistent with episodes of mind-wandering or attentional drift during extended intervals without stimulation (Gouraud et al., 2018). In contrast, the shortening of fixation durations across blocks occurred alongside increased blink frequency and duration as well as reduced eyelid opening, all of which are markers commonly associated with declining vigilance and fatigue. In this case, shorter fixations may reflect less sustained visual processing rather than reduced visual exploration *per se*.

Several mechanisms associated with task engagement may underlie the increase in reaction times and the reduction of oculomotor exploration observed with longer delays. Extended intervals may reduce motor readiness, slowing participants' ability to initiate corrective actions. They may also impair cognitive readiness, as attention is more likely to drift during long periods without task-relevant stimulation, leaving participants less prepared to detect and evaluate system changes when they occur. Although the present study does not isolate the specific mechanism underlying the temporal disengagement we induced in this task, it has important implications: as temporal disengagement increases, operators require more time to assess the autopilot's actions, underscoring the potential risks of disengagement during human–automation interaction.

We would also like to point out that the break offered to the participants halfway through the experiment may have partially restored vigilance, thereby attenuating the effect of time-on-task on behavioral performance. Despite this, clear oculomotor patterns associated with time spent on task were still observed, consistent with a gradual reduction in vigilance. Nonetheless, given the overall lengthy duration of the proposed experiment (around 90 min), possible contribution of fatigue or boredom to the ToT effects cannot be excluded. However, it is worth noting that a slight decrease in RT across blocks was suggested by the posterior estimates. While this effect is small, boredom or fatigue would be expected to induce the opposite effect, with slower RT as time-on-task increases. Therefore, the probability of the present results being due to these factors remains limited.

Finally, logistic classifiers revealed that the number of saccades, fixation duration, and blink duration could predict whether participants would respond slowly or quickly, performing reliably above chance. Together, these findings demonstrate that disengagement meaningfully impacts human performance and that specific oculomotor patterns provide predictive markers of an operator's behavioral state. Beyond their relevance for monitoring piloting activities, these results contribute more broadly to our understanding of how task disengagement affects cognitive functioning. However, the limited ecological validity of the proposed cognitive task requires future studies to validate the present findings using cockpit simulators.

A limitation of the present study is the relatively small sample size, which may constrain the generalizability of the findings. Furthermore, the adopted laboratory paradigm cannot fully capture the extent of vigilance and engagement decrement pilots might experience during long flights. Future research should replicate these effects in larger and more diverse samples, including pilots during flight simulations, to further validate the identified behavioral and oculomotor signatures of disengagement.

## Data Availability

The raw data supporting the conclusions of this article will be made available by the authors upon request, without undue reservation.

## Footnotes

1 Note that due to technical issues, only 101 parameter change were recorded for two participants and 145 for another.
