# Supplementary material for: Distinct oculomotor signatures for task disengagement and reduction in vigilance during a supervisory task
Source: Front Neuroergon. 2026 May 15;7:1706491. doi: 10.3389/fnrgo.2026.1706491 (PMC13219014; doi:10.3389/fnrgo.2026.1706491)
Supplement: Supplementary file 1 [file Data_Sheet_1.docx]

**Supplementary Material for**

**Distinct oculomotor signatures for task disengagement and reduction in vigilance during a supervisory task**

Stefania C. Ficarella^1,2,3^, Nicolas Maille^1^, Nicolas Lantos^1^, Kevin Le Goff^4^, Jean-François Sciabica^4^, Jean-Christophe Sarrazin^1,2,3^, Andrea Desantis^1,2,3^

^1^ *Information Processing and Systems Department, Cognitive engineering and applied neuroscience, ONERA, 13300, Salon de Provence, France*

^2^ *Institut de Neurosciences de la Timone, CNRS & Aix-Marseille Université, Marseille, France*

^3^ *Fédération ENAC ISAE-SUPAERO ONERA, Université de Toulouse, 31000, Toulouse, France*

^4^ *Airbus Operations SAS, Toulouse, France*

**Protocol**

Regarding the significant parameter change to detect, prior to the experiment, we tested several values ranging from 10 to 30 units for changes in altitude, speed, and heading, and from 0.5 to 2 units for changes in flaps. We ultimately selected changes of 20 units and 1 unit, respectively, because they provided a reasonable level of difficulty for identifying signal changes relative to background noise. Changes of 10 units and 0.5 units were too difficult to distinguish from noise, whereas changes of 30 units and 2 units were too easy.

**Bayesian analyses**

All models were estimated using four Markov chains, each run for 4,000 iterations with 1,000 warm-up iterations, yielding a total of 12,000 post-warmup samples per model. We used weakly informative default priors from brms for regression coefficients, intercepts, and variance components. These priors prevent extreme estimates while remaining minimally constraining, ensuring that inferences are primarily driven by the data. To ensure stable sampling, we set a stricter-than-default set of tuning parameters:

- adapt_delta = 0.95, reducing the probability of divergent transitions,
- max_treedepth = 12, allowing exploration of complex posterior geometries.

Model convergence was evaluated using standard diagnostics. Across all models, R̂ values were inferior to 1.01, indicating that chains mixed well and converged reliably. In addition, both Bulk and Tail Effective Sample Sizes (ESS) were sufficiently large (typically well above 1,000), ensuring stable and accurate estimation of posterior means and credible intervals. Posterior predictive checks (pp_check) further confirmed that the models provided adequate fits to the observed data.

**Bayesians tables**

Each table below reports fixed-effects summary for the Bayesian regression models. For each parameter, the table reports the posterior mean (Estimate), posterior standard deviation (Est.Error), and the 95% credible interval (l-95% CI, u-95% CI). Convergence diagnostics are provided via R̂ (values < 1.01 indicate excellent convergence) and the effective sample sizes (Bulk_ESS, Tail_ESS) reflecting the stability of posterior means and interval estimates.

**Table S1. Accuracy**

| Parameter | Estimate | Est.Error | l-95% CI | u-95% CI | Rhat | Bulk_ESS | Tail_ESS |
| --- | --- | --- | --- | --- | --- | --- | --- |
| (Intercept) | 2.283 | 0.203 | 1.892 | 2.693 | 1.001 | 3397.274 | 4344.997 |
| Delay | -0.004 | 0.003 | -0.01 | 0.003 | 1.001 | 19055.078 | 8626.83 |
| Block | 0.015 | 0.03 | -0.044 | 0.074 | 1 | 14519.037 | 8748.512 |
| RT | -0.044 | 0.022 | -0.087 | 0.001 | 1 | 11730.027 | 8531.125 |

**Table S2. Response times**

| Parameter | Estimate | Est.Error | l-95% CI | u-95% CI | Rhat | Bulk_ESS | Tail_ESS |
| --- | --- | --- | --- | --- | --- | --- | --- |
| (Intercept) | 3.616 | 0.275 | 3.07 | 4.156 | 1.001 | 2037.609 | 3318.051 |
| Delay | 0.003 | 0.001 | 0.001 | 0.005 | 1.001 | 15117.447 | 8502.169 |
| Block | -0.015 | 0.009 | -0.034 | 0.002 | 1.001 | 10470.336 | 6642.337 |
| Accuracy1 | 0.221 | 0.097 | 0.032 | 0.414 | 1.001 | 9476.401 | 7767.578 |

**Table S3. Number of saccades**

| Parameter | Estimate | Est.Error | l-95% CI | u-95% CI | Rhat | Bulk_ESS | Tail_ESS |
| --- | --- | --- | --- | --- | --- | --- | --- |
| (Intercept) | 1.809 | 0.118 | 1.578 | 2.046 | 1.001 | 1700.761 | 2442.483 |
| Delay | -0.001 | 0 | -0.002 | 0 | 1.001 | 12813.246 | 7106.763 |
| Block | -0.003 | 0.004 | -0.011 | 0.005 | 1 | 8598.791 | 7485.192 |

**Table S4. Number of fixations**

| Parameter | Estimate | Est.Error | l-95% CI | u-95% CI | Rhat | Bulk_ESS | Tail_ESS |
| --- | --- | --- | --- | --- | --- | --- | --- |
| (Intercept) | 2.032 | 0.088 | 1.859 | 2.207 | 1.003 | 1109.316 | 1884.897 |
| Delay | -0.001 | 0 | -0.002 | 0 | 1.001 | 11795.031 | 7713.844 |
| Block | 0.003 | 0.003 | -0.003 | 0.009 | 1.001 | 8025.94 | 7546.123 |

**Table S5. Duration of fixations**

| Parameter | Estimate | Est.Error | l-95% CI | u-95% CI | Rhat | Bulk_ESS | Tail_ESS |
| --- | --- | --- | --- | --- | --- | --- | --- |
| (Intercept) | 1.191 | 0.016 | 1.158 | 1.222 | 1.002 | 1626.846 | 1955.359 |
| Delay | 0 | 0 | 0 | 0.001 | 1 | 11748.23 | 7756.562 |
| Block | -0.007 | 0.001 | -0.009 | -0.005 | 1 | 19136.503 | 7878.835 |

**Table S6. Number of blinks**

| Parameter | Estimate | Est.Error | l-95% CI | u-95% CI | Rhat | Bulk_ESS | Tail_ESS |
| --- | --- | --- | --- | --- | --- | --- | --- |
| (Intercept) | -0.219 | 0.16 | -0.545 | 0.095 | 1.002 | 1656.586 | 2691.988 |
| Delay | -0.002 | 0.001 | -0.004 | 0 | 1 | 18467.3 | 7877.355 |
| Block | 0.055 | 0.009 | 0.038 | 0.072 | 1 | 8098.127 | 7082.901 |

**Table S7. Duration of blinks**

| Parameter | Estimate | Est.Error | l-95% CI | u-95% CI | Rhat | Bulk_ESS | Tail_ESS |
| --- | --- | --- | --- | --- | --- | --- | --- |
| (Intercept) | -0.807 | 0.076 | -0.961 | -0.657 | 1.001 | 2056.374 | 3309.261 |
| Delay | -0.001 | 0.001 | -0.002 | 0.001 | 1 | 12142.127 | 8033.555 |
| Block | 0.022 | 0.006 | 0.011 | 0.034 | 1.001 | 10682.791 | 8110.37 |

**Table S8. Eyelid opening**

| Parameter | Estimate | Est.Error | l-95% CI | u-95% CI | Rhat | Bulk_ESS | Tail_ESS |
| --- | --- | --- | --- | --- | --- | --- | --- |
| (Intercept) | -4.437 | 0.047 | -4.534 | -4.345 | 1.004 | 1159.922 | 1712.097 |
| Delay | 0 | 0 | 0 | 0 | 1.001 | 11832.852 | 7659.928 |
| Block | -0.002 | 0.001 | -0.004 | -0.001 | 1.001 | 12309.086 | 8488.358 |

**Posterior predictive checks**

Posterior predictive checks for all Bayesian models. Each figure below shows the observed data (black) overlaid with 100 simulated data drawn from the posterior predictive distribution (light blue).

**
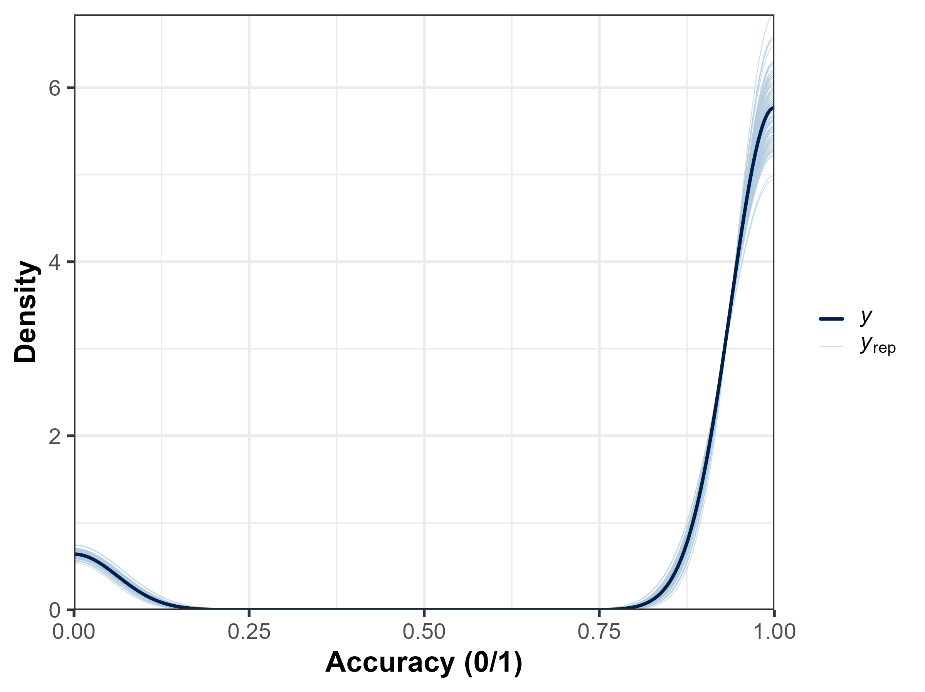
**

Figure S1. Accuracy


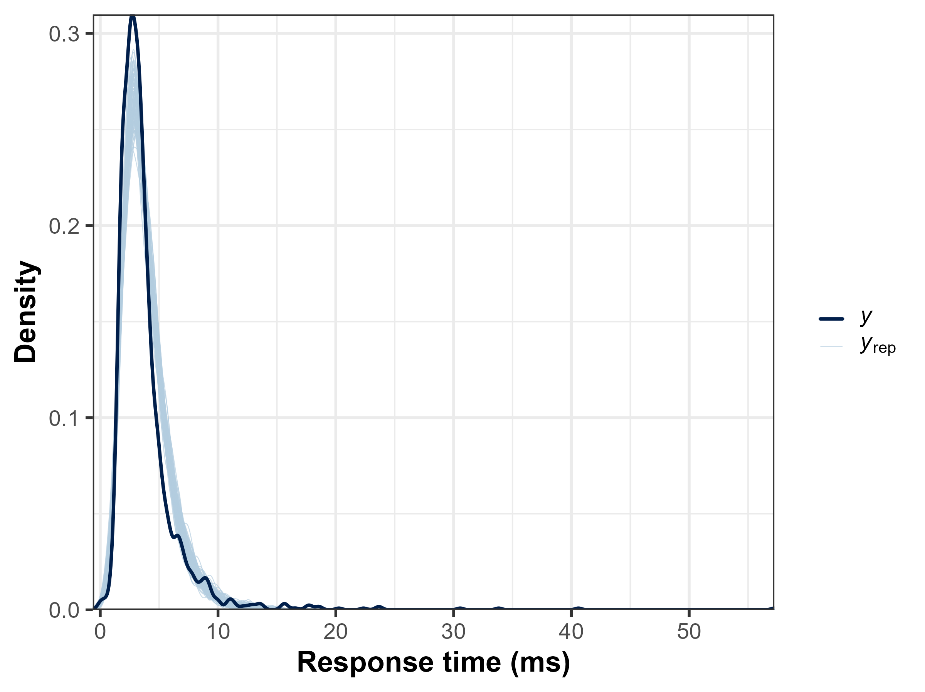


Figure S2. Response times


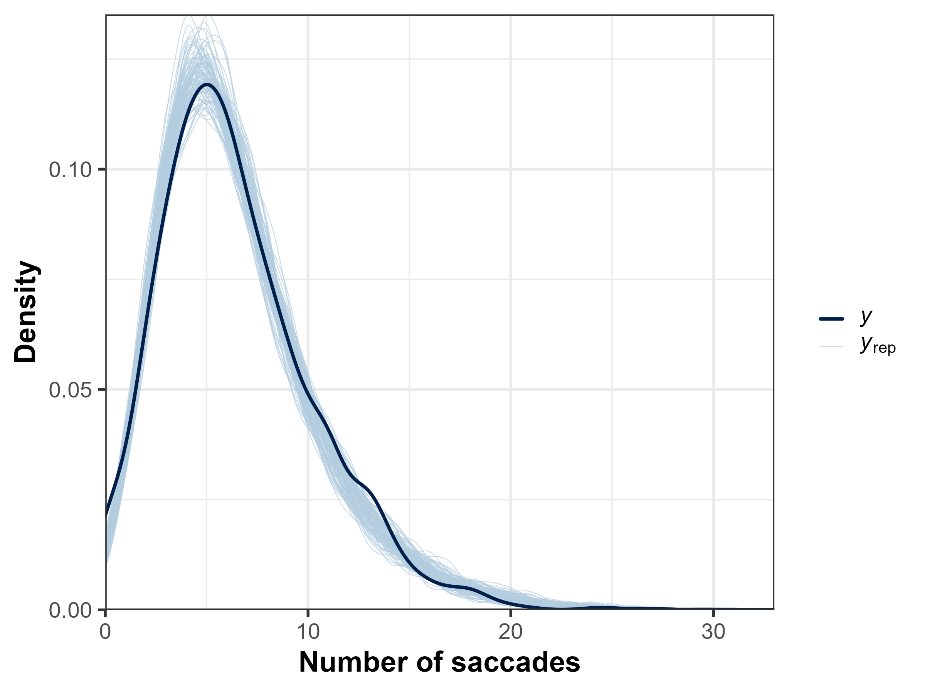


Figure S3. Number of saccades


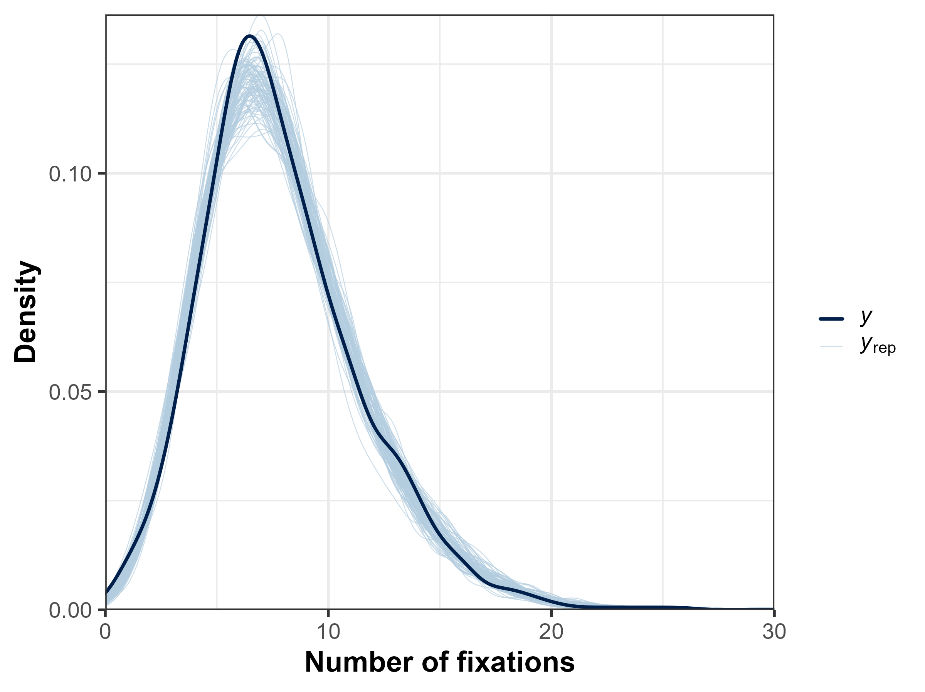


Figure S4. Number of fixations


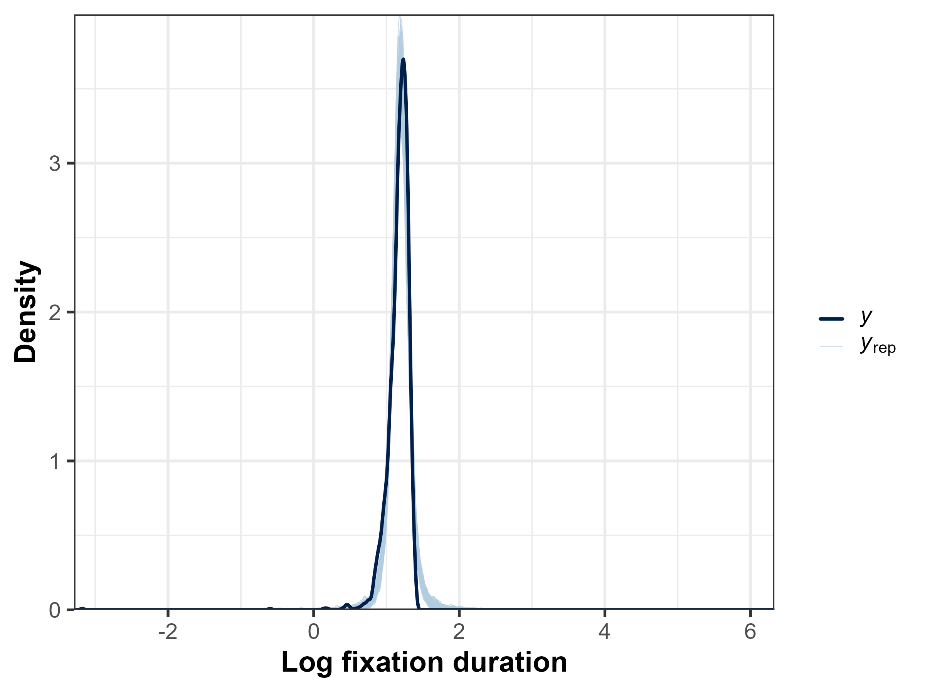


Figure S5. Fixation duration


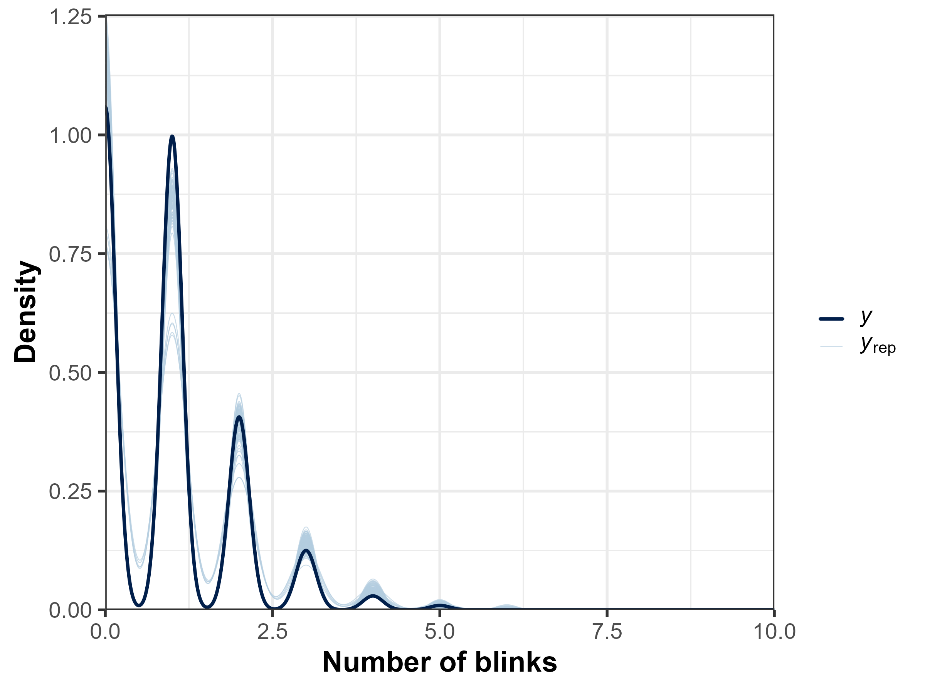


Figure S6. Number of blinks


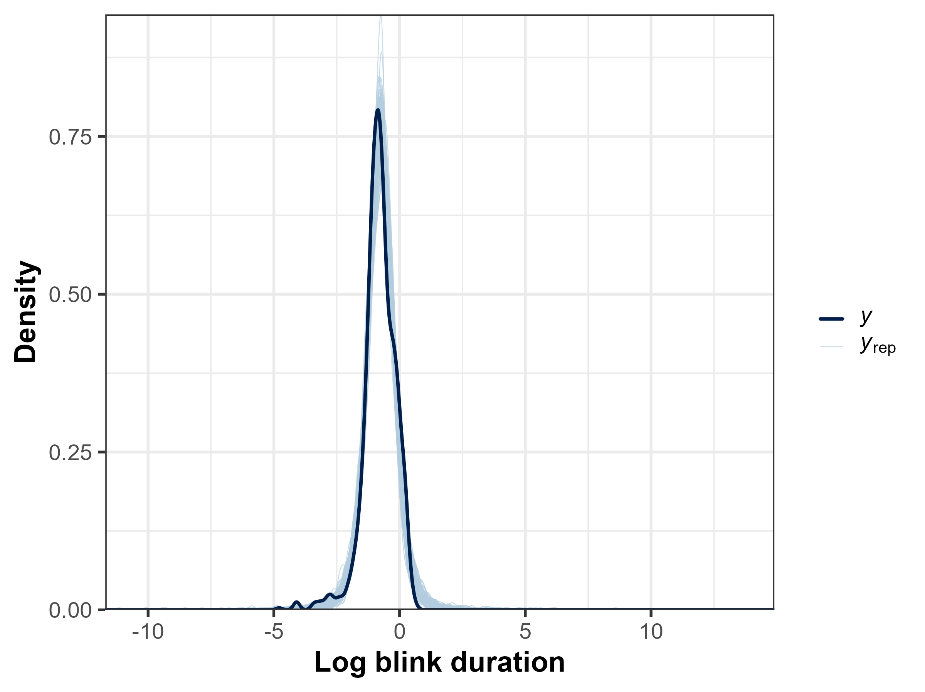


Figure S7. Blink duration


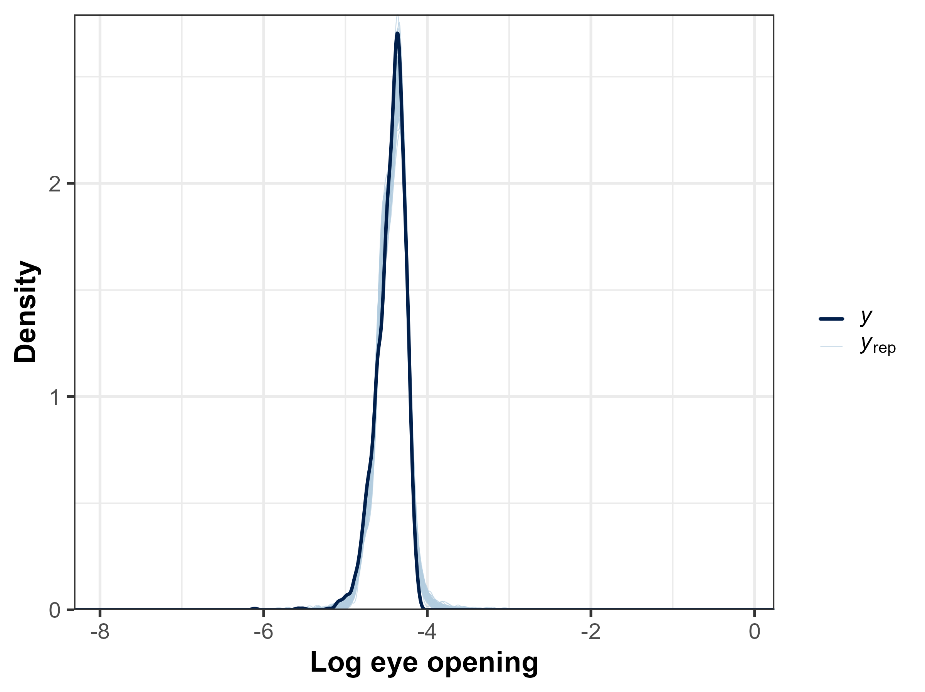


Figure S8. Eyelid opening

**Model comparison and results with random slopes**

We explored models including by-participant random slopes. However, given the relatively small number of participants, estimating random slope variance components is challenging and may lead to unstable or poorly identified models. In such situations, the data provide limited information to reliably estimate between-participant variability in slopes. Hence, we compared models fitting response times using leave-one-out cross-validation. Specifically, we compared three models. The simplest model included only by-participant random intercepts (rt ~ delay + block + accuracy + (1 | participant)). A more complex model included by-participant random intercepts and slopes for all predictors (rt ~ delay + block + accuracy + (1 + delay + block + accuracy | participant)). The comparison indicated that the simpler model had slightly better predictive performance than the more complex model (ΔELPD = −7.6, SE = 7.2), although the difference was within the standard error and therefore did not provide clear evidence favoring either model.

We then compared the simpler model to an intermediate model that included random slopes only for the experimentally manipulated factors delay and block (rt ~ delay + block + accuracy + (1 + delay + block | participant)). This comparison showed that the model including random slopes for delay and block did not meaningfully improve predictive performance relative to the simpler model with only by-participant random intercepts (ΔELPD = 0.1, SE = 4.8), while substantially increasing model complexity. For these reasons, and following the principle of parsimony, we retained the simpler model with by-participant random intercepts in the main analyses.

Regarding the oculomotor variables, the results after including random slopes were mixed. In some cases, models including random slopes produced substantial sampling pathologies (divergent transitions and poor energy diagnostics), indicating that the model parameters were not reliably identifiable given the number of participants. This was observed with the number of fixations and the number of blinks. In other cases when no specific fitting issue was observed, the results obtained with or without random slopes were very similar. This was the case with the number of saccades, the fixation duration, and blink duration. Consequently, for simplicity and parsimony we retained the simpler random-intercept specification, which showed overall good convergence diagnostics and stable posterior estimates. Only for eye opening we observed improved fitting when including random slopes and the disappearance of the block effect that was instead present in the analyses with only random intercepts.

We report below the tables for the models including random slopes but no sampling pathologies.

**Table S9.** Including random slopes improved model fitting of the number of saccades. Saccade number ~ delay + block + (1 + delay + block | subject). The results remained similar to the analyses with random intercepts only.

| Parameter | Estimate | Est.Error | Q2.5 | Q97.5 |
| --- | --- | --- | --- | --- |
| Intercept | 1.81137711 | 0.11801281 | 1.57763895 | 2.05198896 |
| delay_centered | -0.0011422 | 0.00058093 | -0.0023253 | -3.01E-05 |
| block_centered | -0.0057098 | 0.00978903 | -0.0252745 | 0.01378698 |

**Table S10**. Including random slopes improved model fitting of fixation duration. Fixation duration ~ delay + block + (1 + delay + block | subject). The results remained similar to the analyses with random intercepts only.

| Parameter | Estimate | Est.Error | Q2.5 | Q97.5 |
| --- | --- | --- | --- | --- |
| Intercept | 1.19112384 | 0.01737402 | 1.15671911 | 1.22573359 |
| delay_centered | 0.00029985 | 0.00012157 | 6.17E-05 | 0.00054304 |
| block_centered | -0.0067374 | 0.00246686 | -0.0116362 | -0.0017996 |

**Table S11**. Including random slopes improved model fitting of eye opening data. Eye opening ~ delay + block + (1 + delay + block | subject). However, the effect of block disappeared, suggesting that large variability across subjects, thus making the group effect of block on eye opening uncertain.

| Parameter | Estimate | Est.Error | Q2.5 | Q97.5 |
| --- | --- | --- | --- | --- |
| Intercept | -4.4374669 | 0.05263574 | -4.5443178 | -4.334433 |
| delay_centered | -0.0001183 | 0.0001444 | -0.0004126 | 0.00016448 |
| block_centered | -0.0019893 | 0.00252018 | -0.006967 | 0.0029927 |

**Table S12**. Including random slopes did not improve model fitting of blink duration, it actually made it worse (ΔELPD = -0.6, SE = 2.1), although the difference was within the standard error and therefore did not provide clear evidence favoring either model. Blink duration ~ delay + block + (1 + delay + block | subject). However, the results remained similar to the analyses with random intercepts only.

| Parameter | Estimate | Est.Error | Q2.5 | Q97.5 |
| --- | --- | --- | --- | --- |
| Intercept | -0.8091345 | 0.07916821 | -0.9715505 | -0.6522675 |
| delay_centered | -0.000829 | 0.00083427 | -0.0025058 | 0.00075804 |
| block_centered | 0.02414993 | 0.00835419 | 0.0081712 | 0.04157694 |

**Behavioral results: descriptive statistics**

The figures below depict average accuracy for each participant as a function of delay (Figure S9) and block (Figure S10).
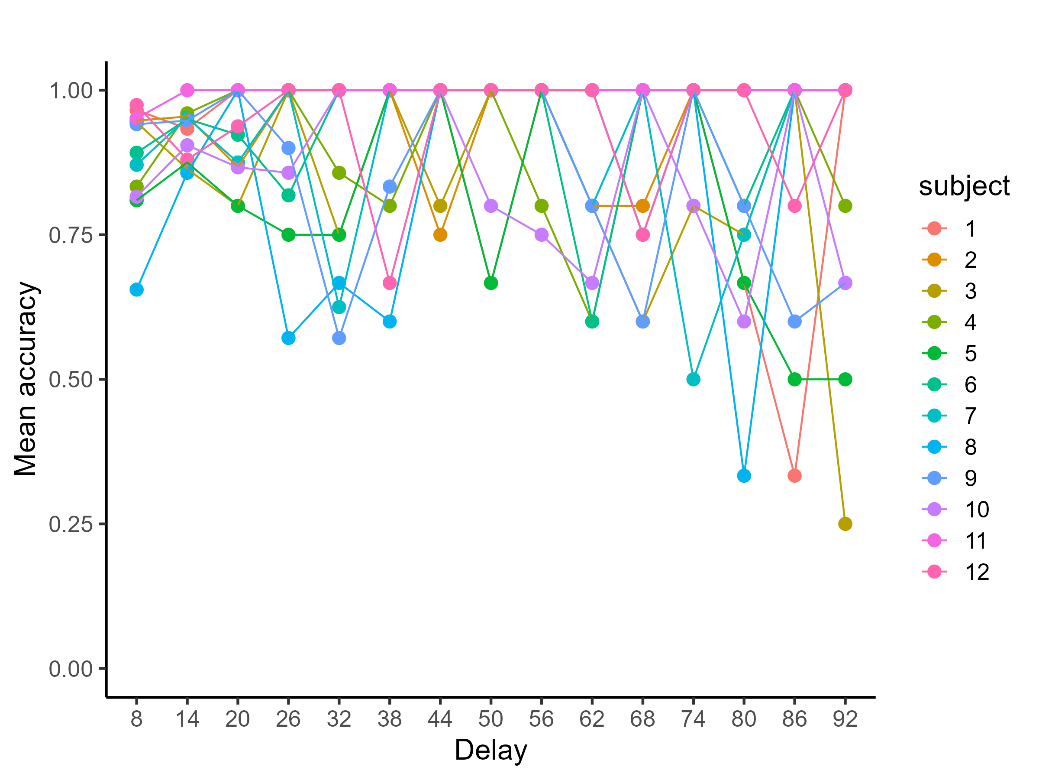


Figure S9. Average accuracy for each participant as a function of delay.


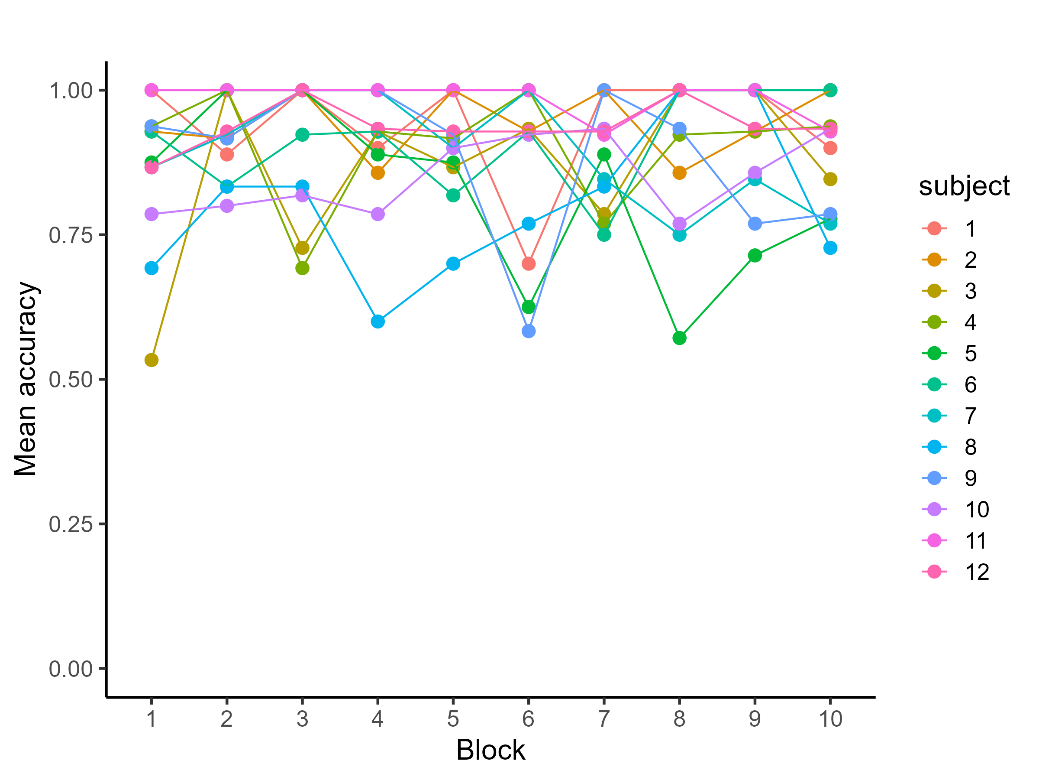


Figure S10. Average accuracy for each participant as a function of block.

**Classification analyses**

The figures below show ROC curves for classifiers predicting response times from eye features observed either within a single 4-second time window preceding the parameter change (Figure S11) or within three successive 2-second time windows preceding the parameter change (Figure S12).


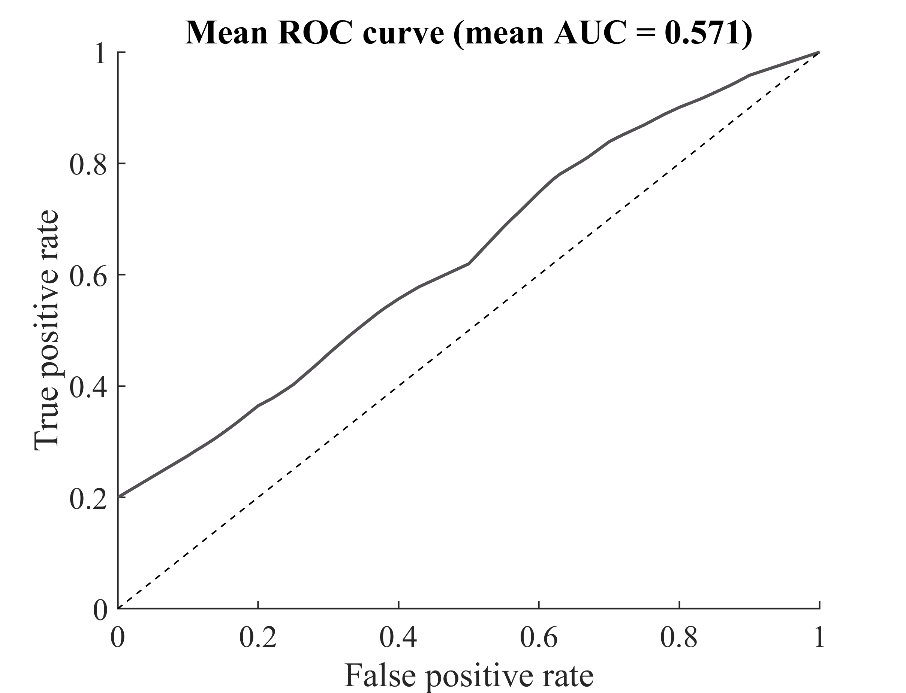


Figure 11S. ROC Curve for 4-second time window classifier.


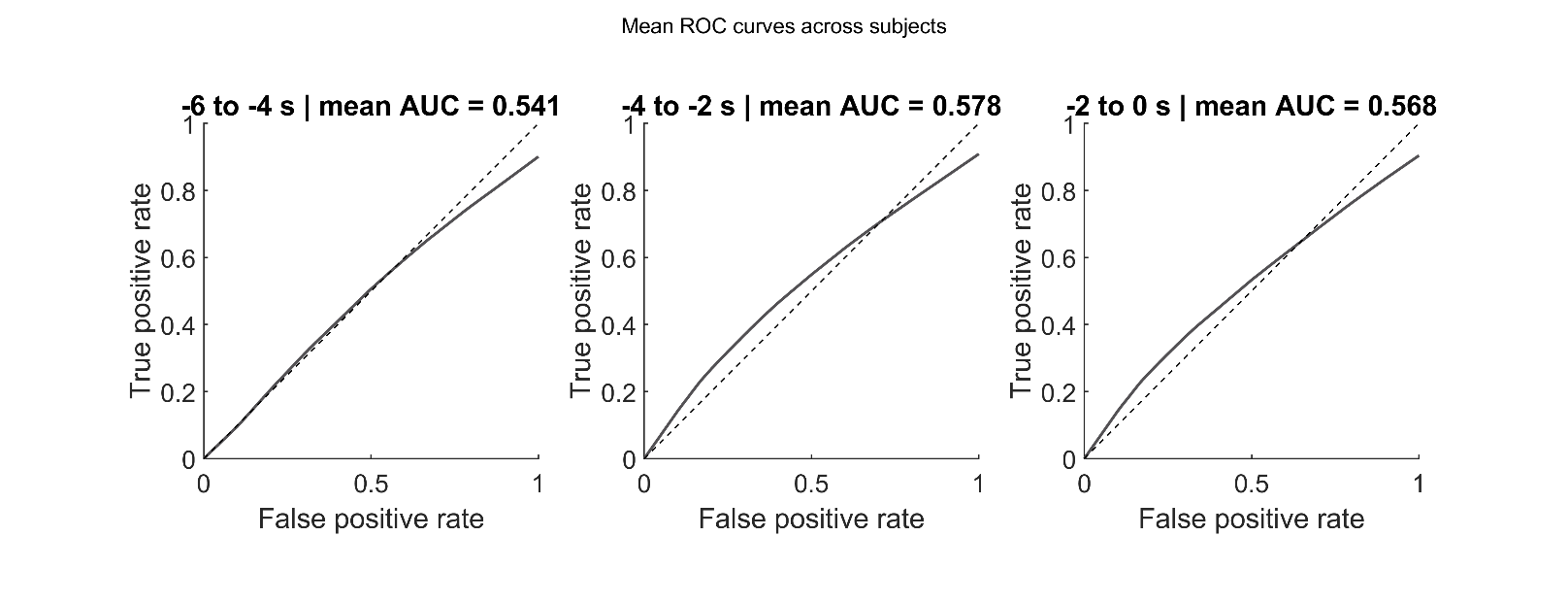


Figure S12. ROC Curve for each 2-second time windows of the original classifier
